# Acoustophoresis of monodisperse oil droplets in water: Effect of symmetry breaking and non-resonance operation on oil trapping behavior

**DOI:** 10.1063/5.0175400

**Published:** 2023-12-27

**Authors:** H. Bazyar, M. H. Kandemir, J. Peper, M. A. B. Andrade, A. L. Bernassau, K. Schroën, R. G. H. Lammertink

**Affiliations:** 1 Engineering Thermodynamics, Process & Energy Department, Faculty of Mechanical, Maritime and Materials Engineering, Delft University of Technology, Leeghwaterstraat 39, 2628CB Delft, The Netherlands; 2 Department of Electrical Engineering and Automation, Aalto University, 02150 Espoo, Finland; 3 Soft Matter Fluidics and Interfaces, MESA+ Institute for Nanotechnology, University of Twente, P. O. Box 217, 7500 AE Enschede, The Netherlands; 4 Institute of Physics, University of São Paulo, São Paulo 05508-090, Brazil; 5 School of Engineering and Physical Sciences, Heriot-Watt University, Edinburgh, United Kingdom; 6 Membrane Processes for Food, University of Twente, P. O. Box 217, 7500 AE Enschede, The Netherlands

## Abstract

Acoustic manipulation of particles in microchannels has recently gained much attention. Ultrasonic standing wave (USW) separation of oil droplets or particles is an established technology for microscale applications. Acoustofluidic devices are normally operated at optimized conditions, namely, resonant frequency, to minimize power consumption. It has been recently shown that symmetry breaking is needed to obtain efficient conditions for acoustic particle trapping. In this work, we study the acoustophoretic behavior of monodisperse oil droplets (silicone oil and hexadecane) in water in the microfluidic chip operating at a non-resonant frequency and an off-center placement of the transducer. Finite element-based computer simulations are further performed to investigate the influence of these conditions on the acoustic pressure distribution and oil trapping behavior. Via investigating the Gor’kov potential, we obtained an overlap between the trapping patterns obtained in experiments and simulations. We demonstrate that an off-center placement of the transducer and driving the transducer at a non-resonant frequency can still lead to predictable behavior of particles in acoustofluidics. This is relevant to applications in which the theoretical resonant frequency cannot be achieved, e.g., manipulation of biological matter within living tissues.

## I. INTRODUCTION

Acoustophoresis is a non-contact and contaminant-free technique used to manipulate or separate particles in a fluid by combining sound waves usually with laminar flow fields and typically in a microfluidic device.^1,2^ This method has broad technological implications including food,^3–5^ pharmaceutical,^6^ biomedical,^7–9^ petrochemical,^10,11^ and oil recovery processes.^12–15,11,16^ Ultrasound has also found its application in energy-efficient emulsion fabrication using cavitation intensifying bags.^17^

Using ultrasonic standing wave (USW) for separation applications is auspicious due to the excellent control of the particle movement and the minimal induced mechanical stress.^8,18,19^ The use of USWs in microfluidics, i.e., acoustofluidics, is a mature technique capable of sorting particles by size or acoustic contrast factor,^20–22^ affinity-specific particle selection and sample de-complexing,^23^ sonocrystallization or emulsification,^24^ free flow transport of cells,^25^ and ultrasonic cavitation.^26,27^ Due to the tunable nature of the acoustic waves and operating frequencies (kHz–GHz), manipulating a wide range of particle sizes (from nm to mm scale) is possible via acoustofluidics.^28^ This unique characteristic opens application perspectives in diverse fields including separation,^20,22,21^ crystallization and emulsification,^24^ and ultrasonic cavitation.^26,27^ Acoustofluidics have attracted significant attention in clinical applications and the biomedical field, where label-free and non-contact particle manipulation is needed. Examples include cell manipulation for single-cell analysis, bioparticle isolation for diagnostics, workflow automation in life science laboratories, cell and gene therapy, tissue engineering, and 3D cell culture.^29^

A typical acoustofluidic setup consists of a microchannel (chamber), function generator, power amplifier, and the piezoelectric transducer as the core component, which converts electrical signals to mechanical strain.^28,30^ The transducer is driven by a sinusoidal signal, and the resulting, preferably resonant, harmonic response of the chamber leads to the formation of ultrasonic standing waves (USWs). In a typical USW, pressure nodes form at regions where the pressure gradient is maximum, and anti-nodes form where the gradient is minimum.

The working principle of acoustophoresis is based on the effects of acoustic radiation forces on particulate matter. When USWs are generated in a medium containing particles or droplets, the waves will be scattered if there is acoustic impedance contrast (difference in acoustic impedance) between the fluid and the particle/droplet.^19^ The scattering of the wave produces a primary acoustic radiation force that transports the particles toward the acoustic pressure node or anti-node according to the density and compressibility of the particles and the surrounding medium.^22,19^ Although the acoustic radiation force is mostly employed for trapping and manipulating spherical particles with a size much smaller than the acoustic wavelength, there are also numerous theoretical and experimental studies describing its use for trapping, translating, and rotating larger non-spherical objects, such as ellipsoids, cones, and diamonds.^31–35^

Over the last decades, various materials, geometrical designs, and strategies for ultrasonic actuation have been implemented to develop acoustofluidic devices. Despite the differences, they are all designed to operate under optimized states to minimize power consumption and maximize the focusing ability of acoustic fields.^28,36^ The USW usually has a pressure nodal plane parallel to the sidewalls of the channel and in the middle of the channel. The actuation frequency is tuned to generate half-wavelength resonators with a pressure node in the center and anti-nodes at the channel sides (see the upper inset in Figure 2). This leads to precise movement and manipulation of particles.^37^ Thus, the best working frequency at which the microchannel is in resonance (resonant frequency) should be defined precisely beforehand.^36,38^

Theoretically, the resonant frequency of a microchannel is determined using the 1D standing planar acoustic wave approximation,

$$f=\frac{c}{2w}n,$$ (1)

where w is the channel width, c is the speed of sound in the fluid, and n is a positive integer, where n=1 corresponds to a half-wavelength resonance inside the channel. In practice, this theoretically calculated frequency may differ from the actual resonant frequency of the microchannels. Various experimental and characterization methods have been developed to determine the optimal working frequency, among which electrical impedance is the most promising and straightforward method.^39,36,38^

A typical 1D USW has an antisymmetric acoustic field with acoustic pressure nodal planes parallel to the channel wall. Much effort has been put into finding the most efficient conditions for acoustic particle trapping. In a numerical study, Ley *et al.* investigated a generic glass capillary excited by a piezoelectric transducer in a symmetric setting.^40^ The work investigates localized resonances for different capillary geometries. The results indicate that this excitation can only generate acoustic pressure nodal planes parallel to the transducer surface. In contrast, the acoustic pressure distribution in other directions remains symmetric. Symmetry breaking is necessary to obtain nodal planes perpendicular to the transducer, as implied by earlier works in which the channel walls are assumed to be excited non-symmetrically.^8,41,42^ Later works introduce the whole-system ultrasound resonance concept by including every physical part in numerical studies to identify the optimal conditions for acoustic particle trapping.^36,38^ Despite the symmetric system, symmetry breaking was necessary to obtain an antisymmetric acoustic pressure field in the channel. The electrodes of the transducer were actuated anti-symmetrically. It has been found that acoustic trapping of particles was obtained at frequencies below half wavelength resonance. In contrast, the frequency of maximum focusing ability still coincided with the resonant frequency of the structure, i.e., frequency of the maximum admittance. More recently, asymmetric geometries have been studied numerically and experimentally, demonstrating that stronger particle trapping fields and meaningful particle aggregation times can be obtained via exciting asymmetric chip structures.^43^ This has a significant impact specifically for manipulating particles with low acoustic contrast factors. A recent study^44^ demonstrated that the ultimate symmetry breaking by actuating the channel directly from the side is superior to other actuation schemes.

Despite the recent advances in acoustofluidic devices, there is a need for information on their operation in non-resonant frequency and sub-optimal placement of the transducer. In this work, we investigate how an off-center placement of the transducer and driving the transducer off resonance affect the acoustophoretic behavior of oil droplets in water. To reduce the effects of symmetry breaking, we investigate the effect of the slightly off-center location of the transducer on the acoustic field and the movement of oil droplets under these conditions. Experiments are performed to investigate acoustic trapping of monodisperse oil-in-water (O/W) emulsions in a microfluidic channel, operating off resonance and with the transducer slightly displaced from the chip center. Computer simulations based on the finite element method are further carried out to investigate the influence of these conditions on the acoustic pressure distribution and trapping behavior. Section II presents the methods for fabricating monodisperse oil droplets and the acoustophoretic experiments in a microfluidic channel. Section III describes the 2D and 3D computer simulations of the microfluidic chip. Section IV compares the oil droplet behavior in experiments to the predictions from computer simulations. Finally, potential impacts of symmetry breaking and non-resonance operation of the microfluidic chip are discussed.

## II. EXPERIMENTS

### A. Fabrication of monodisperse O/W emulsions

**FIG. 1.**
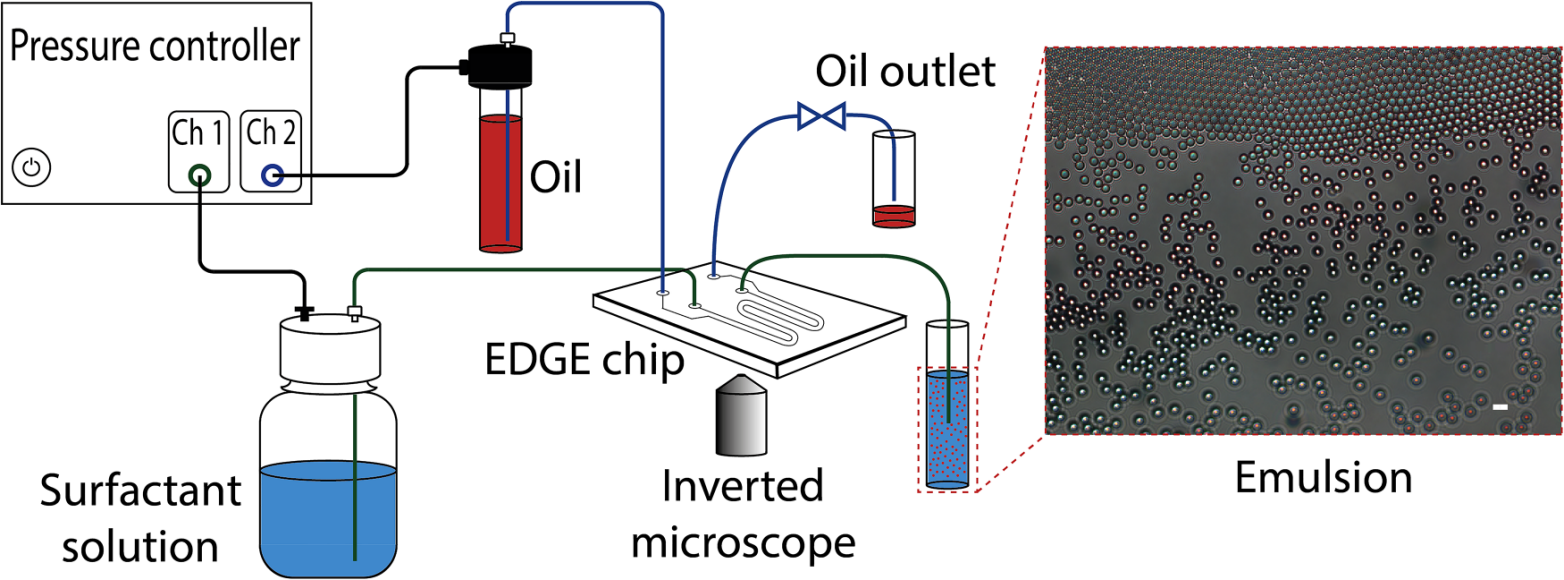
Schematic illustration of monodisperse O/W emulsion fabrication using an EDGE chip and the optical microscopy picture of the prepared HD droplets in SDS solution with a concentration of 62% CMC (SDS62) (scale bar is 20 μm).

Monodisperse oil droplets were used in this study to avoid the statistical uncertainties arising from the polydispersity of particles in the estimation of acoustic energy density.^45^ Monodisperse O/W emulsions were prepared using a microfluidic chip called edge-based droplet generation (EDGE).^46^ The schematic of the setup is shown in Fig. 1. A pressure controller (OB1 Mk3+ from ElveFlow, France) was connected to the nitrogen gas line at 3 bar. Channels 1 and 2 are the low and high-pressure channels that can be set to a maximum of 200 and 2000 mbar, respectively. Channel 1 was connected to the sodium dodecylsulfate (SDS) surfactant solution bottle and pressurized at 10 or 20 mbar. The oil bottle was connected to the high-pressure channel (channel 2) and pressurized at 190–680 mbar depending on the oil type and concentration of SDS in the surfactant solution (see Table S1 in the supplementary material). The oil outlet was closed using a two-way valve during emulsion fabrication to establish the corresponding pressure on the oil channel. The mechanism of droplet formation is explained in detail elsewhere.^47^ The chip is designed in the group of food process engineering at Wageningen University and Research, Wageningen, the Netherlands. It is made out of glass by Micronit Microtechnologies B.V., Enschede, the Netherlands. Anionic SDS was used as the water-soluble surfactant. Four surfactant solutions were prepared by dissolving 10%, 50%, 62%, and 100% of the corresponding critical micelle concentration (CMC) in pure water (Milli-Q grade). From now on, the surfactant solutions are named SDS10, SDS50, SDS62, and SDS100, where the number corresponds to the concentration of SDS as a percentage of the CMC. The CMC of SDS was experimentally measured as 8.1 mM at 20–25 ${}^{{}^{\circ}}$C.^48^ More than 12 h was given for the surfactant to dissolve in water completely. Both hexadecane (HD) and silicone oil (SO) were used as the oil phase. In total, eight monodisperse O/W emulsions were fabricated. The emulsions prepared with SDS62 were used only for the first preliminary experiments and not for further analysis. See Figure S1 and Movie S1 in the supplementary material for the droplet fabrication picture and video, respectively. Figure S2 and Table S2 in the supplementary material show the droplet size distribution and the average droplet size together with the corresponding coefficient of variation (CV) for all the prepared emulsions using SDS10, SDS50, and SDS100.

### B. Acoustic experiments

**FIG. 2.**
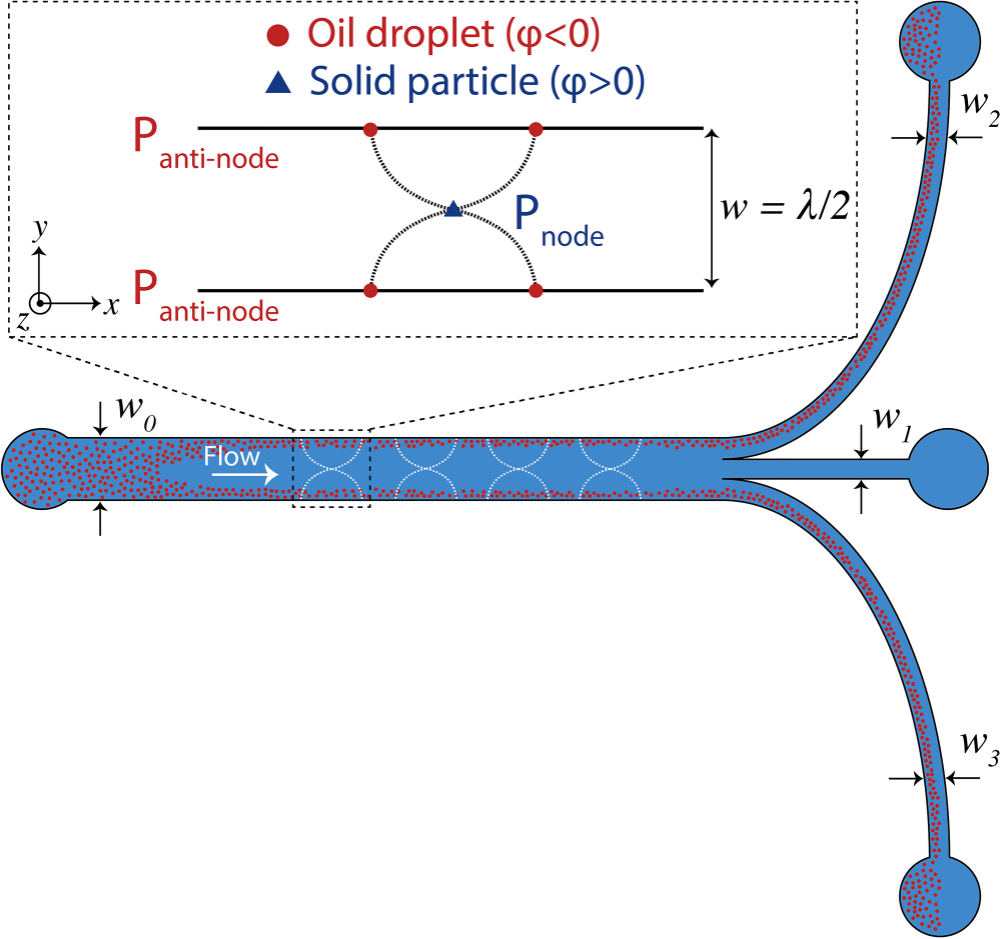
Schematic illustration of the microfluidic chip with one inlet and three outlets. w ${}_{0}$ is 600 μm and w ${}_{1-3}$ is 200 μm. A 1D USW is illustrated in the channel, where particles with a positive ACF (solid particles) move toward pressure nodes and particles with a negative ACF (oil droplets) move toward anti-nodes.

A microfluidic chip with one inlet and three outlets was used to investigate the acoustophoretic behavior of oil droplets (see Fig. 2 for the schematic illustration of the chip). The chip is fabricated in silicon using standard photolithography and reactive ion etching (see section “Fabrication of the microfluidic chip” in the supplementary material for detailed fabrication procedure).

**FIG. 3.**
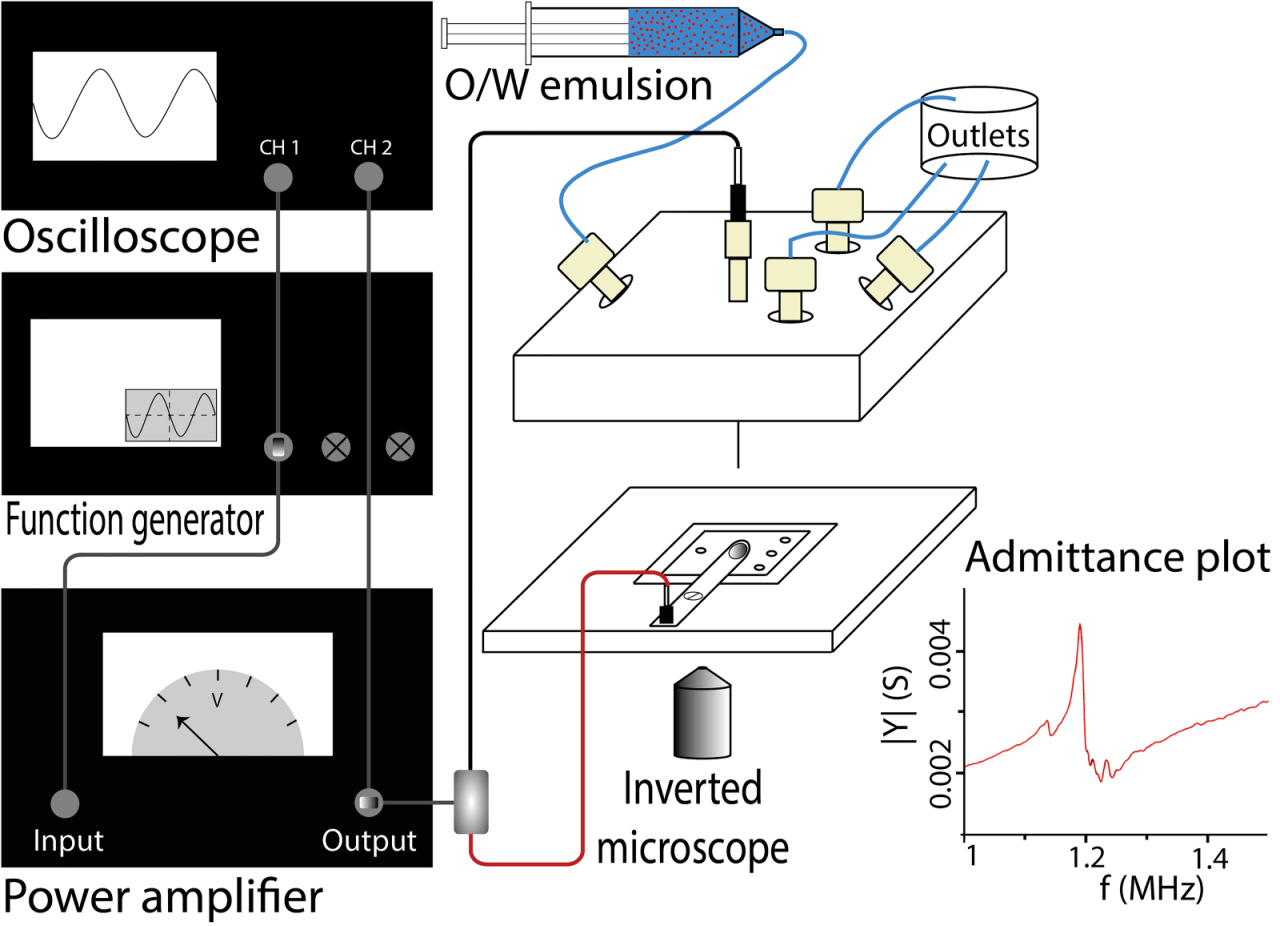
Schematic illustration of the acoustic setup together with the admittance plot of the chip.

The chip was placed in the chip holder. A ceramic piezoelectric transducer [Disk of Pz26 (NavyI), hard relaxor-type PZT with diameter 5 mm and thickness 0.5 mm from Meggitt ferroperm, Denmark] was attached on the backside of the chip using a copper tab ( 28.5×6 mm) fastened to the chip holder using a screw. The piezoelectric transducer (PZT transducer) was soldered on top of the copper tab using standard PbSn solder paste. The connection for the negative polarity was an electronic header pin soldered at the end of the tab [see Figure S7(a) in the supplementary material]. The connection for the positive polarity was custom-built by mounting a spring contact probe with a round tip (SS-50-J-2.9-G with receptacle Rss-50-SC from Mouser electronic, the Netherlands) in a nanotight fitting (F-130 from IDEX health and science, the Netherlands) using an appropriate sleeve to clamp the spring contact in place. On the back of the spring contact probe, a 1-mm plug (SLS1-S from Stäubli Benelux, Belgium) was soldered. The whole connection was then screwed to the top part of the chip holder. Finally, the appropriate socket parts connected wires to the positive (1-mm plug) and negative (header pin) connections. The spring contact probe was in direct contact with the PZT transducer upon closing off the chip holder. The schematic illustration of the acoustic setup is shown in Fig. 3. Since no glue was used to attach the transducer to the chip, the chip and the transducer could be easily re-used.

For the actuation of the PZT transducer, a signal of 200 mV ${}_{\;\mathrm{pp}}$ was generated using a function generator (Tektronix AFG 2021) and amplified up to 20 V ${}_{\;\mathrm{pp}}$ by a power amplifier (EIN 350L RF power amplifier), while the actuation was monitored by an oscilloscope (Tektronix TDS 2022B). The assembled chip’s admittance spectrum is measured using a Gain-phase analyzer (HP 4194A Impedance/Gain-Phase Analyzer) by attaching the measurement probes to the connections on the chip. The inset in Fig. 3 illustrates the admittance spectrum of the chip, where there is a local minimum at 1.25 MHz and the maximum admittance is measured at 1.18 MHz. For investigating the chip behavior under a non-resonant condition, the excitation frequency is set to 1.25 MHz.

All the observations were performed using an inverted optical microscope (Zeiss Axiovert 40 MAT) and objective with a magnification of 10x (Zeiss EC Epiplan 10x/0.25 M27 [free working distance (FWD) =11.0 mm)]. Oil droplets’ acoustophoretic movement was recorded through a camera (Hamamatsu orca flash 4.0 c11440 ) and software (Olympus cellSens Dimensions).

## III. COMPUTER SIMULATIONS

The computer simulations were carried out via COMSOL Multiphysics 6.0 using the computer resources within the Aalto University School of Science “Science-IT” project. Two different sets of computer simulations were carried out.

**FIG. 4.**
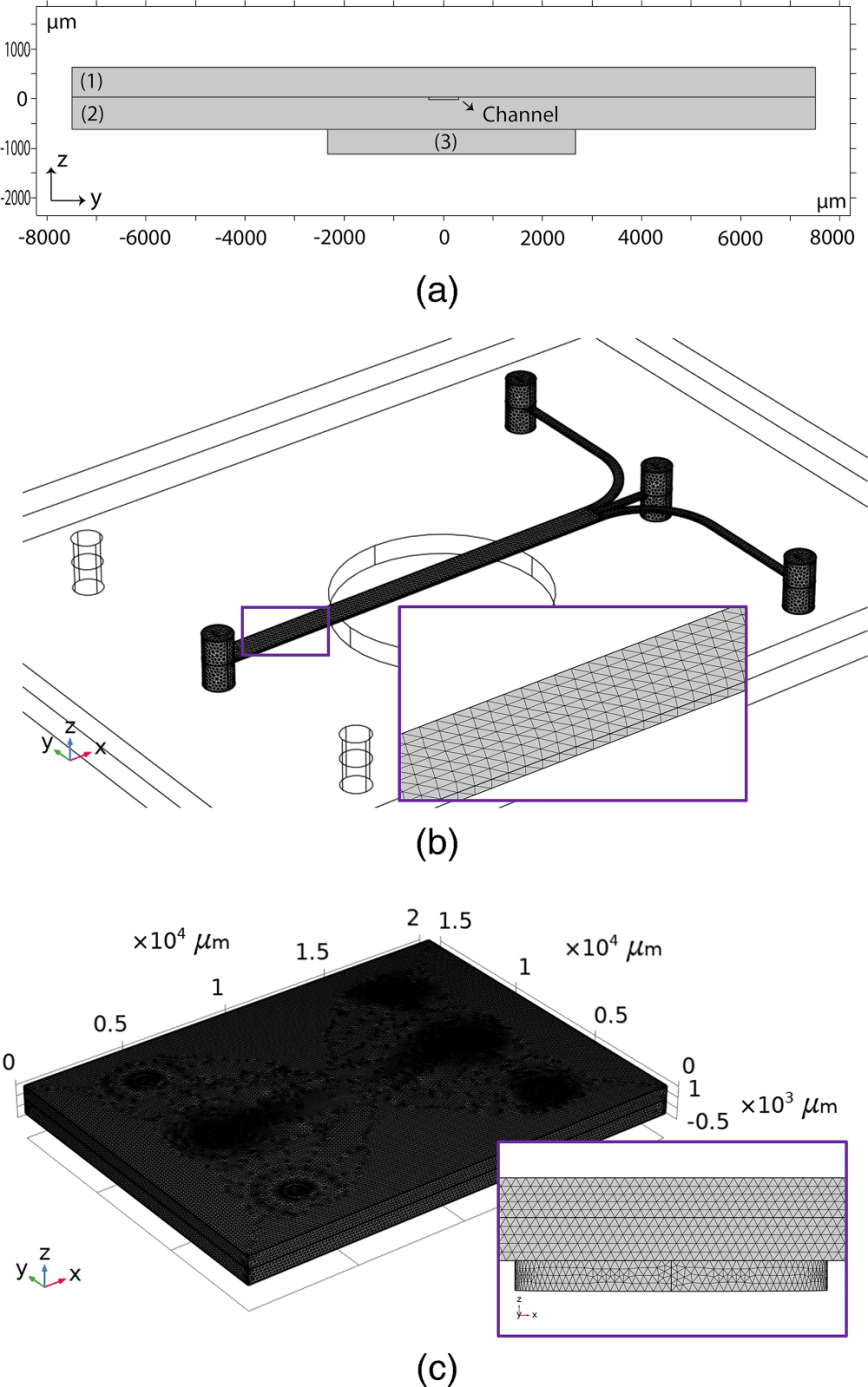
(a) Simplified geometry used in 2D simulations [(1) top glass, (2) silicon substrate, and (3) transducer]. Meshed geometries of the (b) channel and (c) the chip made in COMSOL Multiphysics 6.0. The inset in (b) represents the mesh in the channel section, while the inset in (c) gives a zoomed-in side view of the mesh in the chip material and the piezoelectric transducer.

First, simplified 2D simulations were created to investigate the effect of the symmetrical or non-symmetrical placement of the transducer. The simplified geometry was the 2D cross section of the chip (yz-plane, according to Fig. 4), including the channel, the glass structures, and the transducer [Fig. 4(a)]. By default, the software includes all the necessary boundary and compatibility conditions to model the device. The chip’s acoustic field was solved by using narrow region acoustics physics, with the rectangular duct assumption corresponding to the channel dimensions. This is performed to better represent the potential thermoviscous losses in the channel. Subsequently, 3D simulations were used to analyze the acoustic field, including all the chip details.

Before 3D computer simulations, the chip’s geometry was analyzed in more detail using the CAD drawings of the chip. The exact location of the transducer was determined by visual inspection (see section “Determining exact location of transducer” in the supplementary material for details of both, respectively). The geometry is subsequently recreated in COMSOL without excluding minor details such as unused holes on the chip. In addition, the offset location of the transducer breaks the symmetry preventing any use of the symmetry conditions. To make sure that the mesh does not affect the results, a mesh convergence study is further performed. The results are shown and elaborated in section “Mesh convergence analysis” in the supplementary material. The whole geometry includes 2 009 933 elements with an average element quality for the skewness of 0.6633 and volume-vs-circumradius of 0.6826. The number of degrees of freedom was 8 298 584. On average, 450 GBs of RAM was used by 4x Intel Xeon Gold 6148 CPUs running at 2.4 GHz. The mesh for the inner channel and the whole chip is given in Figs. 4(b) and 4(c).

The material properties used in the simulations are given in Table I. In addition to the values given in Table I, built-in properties for water (as medium in the channel), silica glass (as the top part of the chip), and single crystal silicon (as the bottom part of the chip) were used. The values from the manufacturer were used for PZ26 (as the transducer).

**TABLE I.** Used values in the COMSOL simulations.

Property	Value	Description
h_channel_	50 *μ*m	Channel depth
t_channel_	600 *μ*m	Main channel width
l_channel_	10 225 *μ*m	Main channel length
D_tra_	5 mm	Transducer diameter
h_tra_	0.5 mm	Transducer height
l_chip_	20 mm	Entire chip length
w_chip_	15 mm	Entire chip width
D_in_	700 *μ*m	Inlet hole diameter
V_tra_	20 V	Transducer actuation voltage
d_oil_	6 *μ*m	Average oil droplet diameter
x	0.1079 mm	Offset of transducer location in x-dir
y	0.1593 mm	Offset of transducer location in y-dir

### A. Acoustic radiation force on the oil droplets

The glass chip is excited by the piezoelectric transducer, which leads to the formation of an acoustic field inside the microchannel. Similar to the 1D ultrasonic standing waves,^53,19^ the particles or droplets experience an acoustic radiation force. Since this force is conservative, the radiation force acting on a small spherical object of volume V can be described in terms of an acoustic radiation potential $U_{rad}$, known as the Gor’kov potential

$$U_{rad}=V\left(\phi_{1}\frac{1}{2\rho_{0}c_{0}^{2}}<p_{in}^{2}>-\phi_{2}\frac{3}{4}\rho_{0}<v_{in}^{2}>\right),$$ (2)

where the acoustic radiation force exerted on the spherical particle or droplet is quantified by the gradient of the Gor’kov potential. In Eq. (2), $<p_{in}>$ and $<v_{in}>$ are, respectively, the time-averaged incident pressure and velocity field at the center of the object, $\rho_{0}$ and $c_{0}$ are the density and speed of sound of the host medium, and $\phi_{1}$ and $\phi_{2}$ are the monopole and dipole scattering coefficients, respectively. The combination of the scattering coefficients forms the acoustic contrast factor (ACF), which is given by

$$\phi (\rho ,c)=\frac{1}{3}\phi_{1}+\frac{1}{2}\phi_{2}=\frac{\rho +\frac{2}{3}(\rho -\rho_{0})}{2\rho +\rho_{0}}-\frac{1}{3}\frac{\rho_{0}c_{0}^{2}}{\rho c^{2}},$$ (3)

where ρ and c are the density and speed of sound in the object material, respectively. Objects with positive ϕ (solid particles) and negative ϕ (oil droplets) move toward the pressure minima and maxima, respectively (see the upper inset of Fig. 2). The physical properties of the oil phases (HD and SO), such as density, viscosity, and speed of sound, along with the calculated acoustic contrast factors are shown in Table II. The acoustic radiation force on a spherical object is expressed as

$$F_{rad}=-\nabla U_{\mathrm{rad}},$$ (4)

meaning that the objects are trapped where the Gor’kov potential is minimum. As the potential is a function of particle properties through ACF, solid particles are usually trapped at the pressure nodes, while droplets are trapped at the pressure anti-nodes.

**TABLE II.** Physical properties and acoustic contrast factor of hexadecane, silicone oil, and water.

Liquid	Density at 24 ${}^{{}^{\circ}}$C (g/cm^3^)	Absolute viscosity at 20 ${}^{{}^{\circ}}$C (mPa s)	Speed of sound in liquid (m/s)	Compressibility (×10^−10^) (1/Pa)	Acoustic contrast factor *ϕ* (-)
Hexadecane	0.727 ± 0.002	3.2 ± 0.13	1357^49^	7.46 ± 0.02	−1.006 ± 0.002
Silicone oil AR20	1.003 ± 0.002	19.6 ± 0.1	1350^50^	5.47 ± 0.01	−0.223 ± 0.001
Water	1.00 ± 0.001	1.00^51^	1497^52^	4.462 ± 0.005	−

To simulate the Gor’kov potential and the acoustic radiation force acting on the oil droplets, the incident acoustic pressure distribution $p_{in}$ and the incident velocity field $v_{in}$ are simulated in COMSOL and then replaced in Eq. (2) to calculate the Gor’kov potential along the channel. From the Gor’kov potential, the radiation force $F_{rad}$ is calculated using Eq. (4).

## IV. RESULTS AND DISCUSSION

### A. Experimental results

#### 1. Acoustic experiments

**FIG. 5.**
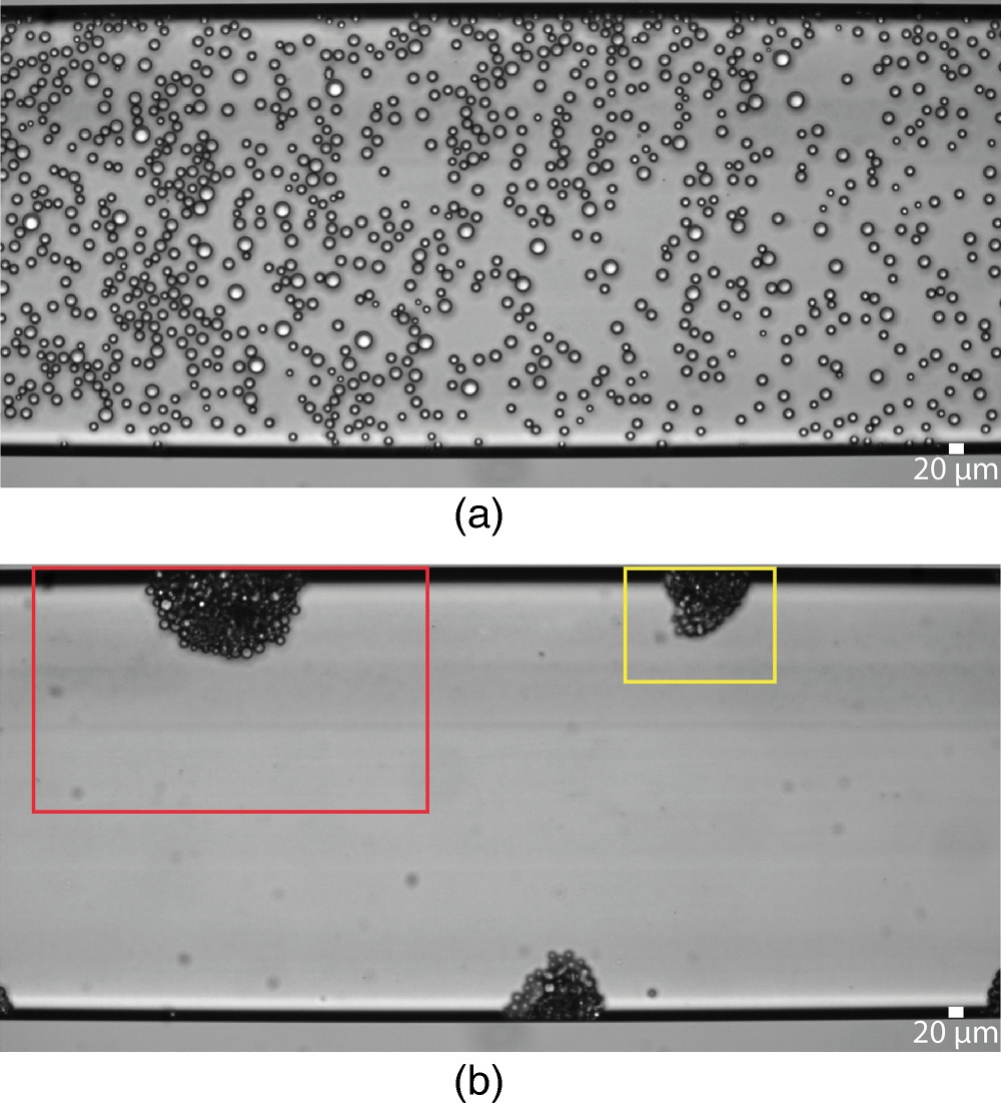
Snapshots of the microchannel at (a) first and (b) 24 th time frame (12 th min) of applying acoustic field at 1.25 MHz and V ${}_{\;\mathrm{pp}}=20$ V for 30 min on SO droplets dispersed in SDS62 solution. The yellow and red rectangles show examples of the field of view for zoomed-in observations at a trapping location (see Fig. 6).

The acoustic experiments were performed on all emulsions (SO and HD droplets in SDS 10, SDS50, SDS62, and SDS100) at 20 V ${}_{\;\mathrm{pp}}$ and a frequency of 1.25 MHz. The acoustophoretic movement of oil droplets was first recorded in the time-lapse mode over the whole channel width for 30 min at two frames per minute (FPM). Figure 5 shows the recorded movie’s first and the 24 th time frames (see Movie S2 in the supplementary material) for concentrated SO droplets in the SDS62 solution. In contrast to a standard acoustofluidic device where the oil droplets would agglomerate along the channel lateral walls, here the oil droplets are trapped at certain spots, with a typical spacing distance close to λ/2 ( $w_{0}$).^18^ This trapping behavior cannot be explained by analytical expressions based on a plane wave, which assume there is a pressure node at the center of the channel and pressure anti-nodes at the lateral walls. Here, the pressure amplitude distribution needs to be evaluated by numerical simulations, and the radiation force acting on each droplet should be calculated by the Gor’kov equation. The pattern of oil droplets at the trapping locations was observed throughout the whole channel, including the outlets. To diminish the particle–particle interaction and ensure the validity of single particle tracking theory,^54^ a more diluted emulsion was used for the rest of the analysis and acoustic experiments of all the other emulsions (see section “Acoustic experimental results” in the supplementary material).

Once the trapping locations were determined, another series of experiments were performed in the zoomed-in mode at different locations. In these experiments, the time-lapse was recorded for 30 s at two frames per second (FPS). As an example, Fig. S3 in the supplementary material illustrates the movies’ first and last time frames for HD and SO droplets in SDS100. Since the speed of sound in water has a significant temperature dependence ( $\frac{\partial c}{\partial T}=4$ m/s K),^55^ the impact of heating from the piezoelectric transducer on the measurements was prevented by actuating it at a moderate power level and for short intervals (5, 10, or maximum 30 s) during all the zoomed-in recordings.

#### 2. Analysis of acoustic experiments

**FIG. 6.**
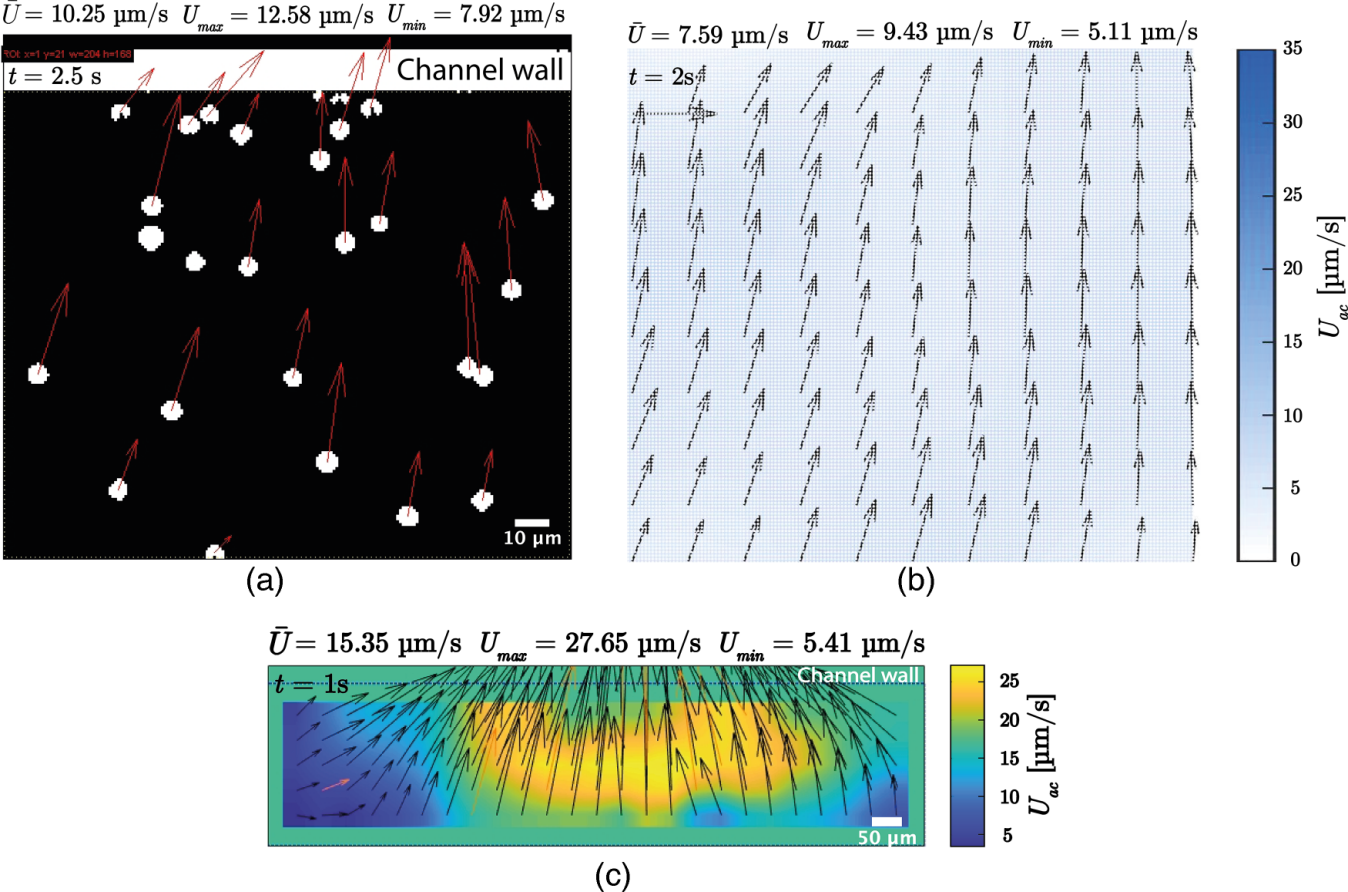
Snapshots of (a) PTV and (b) PIV analyses of the corresponding zoomed-in 5 th time frame [yellow rectangle in Fig. 5(b)]. (c) PIV analysis over half of the channel width for SO in SDS100 showing a velocity field around the anti-node [red rectangle in Fig. 5(b)]. (It is worth nothing that these rectangles show approximate locations of the field of view.)

The acoustophoretic velocity of the oil droplets was measured by tracking the individual oil droplets using particle tracking velocimetry (PTV). The MATLAB tool PTVlab^56^ was used for the corresponding PTV analysis to get the average velocity per frame and velocity vectors (see sections “Particle tracking velocimetry results” and “Acoustophoretic velocity of oil droplets” in the supplementary material for details of PTV and corresponding velocity calculations, respectively). All original images were binarized to get a black background and white droplets using FIJI ImageJ^57^ [see Fig. 6(a) and Movie S4 in the supplementary material for the corresponding video combined with the original movie. A similar video for HD droplets in SDS100 is shown in Movie S5 in the supplementary material]. The acoustophoretic velocity values for different emulsion systems as a function of time and SDS concentration are shown in Figs. S4–S6 in the supplementary material.

To get the localized velocity field around the trapping location, particle image velocimetry (PIV) was performed using the MATLAB tool PIVlab^58^ (see section “Particle image velocimetry results” in the supplementary material for details of PIV analysis). Figure 6(b) shows the snapshots of the 5 th time frame (2.5 s) of the PIV movie for SO droplets in SDS100 solution (see Movies S6 and S7 in the supplementary material for the combined zoomed-in videos with the PIV results of SO and HD droplets in SDS100, respectively). PIV analysis was performed over half the channel width to better understand the velocity field distribution at distances further from the traps. The snapshot of the second time frame (1 s) for SO droplets in SDS100 is shown in Fig. 6(c). The corresponding video combined with the original movie is shown in Movie S8 in the supplementary material.

In this paper, we assume that the droplet’s motion is mainly caused by the primary acoustic radiation force, but the motion can also be affected by the secondary acoustic radiation force and streaming forces. The possible effects of secondary forces and streaming forces on the oil droplet acoustophoresis in the microchannel are explained in the corresponding sections in the supplementary material.

### B. Simulation results

#### 1. 2D simulations

**FIG. 7.**
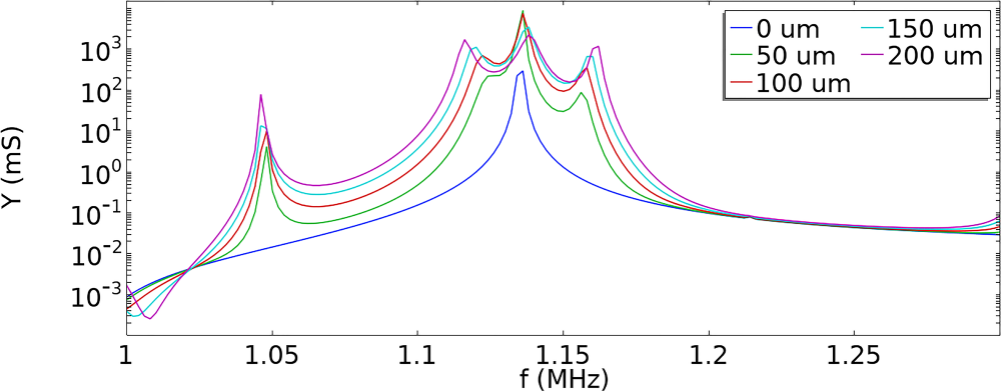
Admittance of the transducer for different offset values in 2D simulations.

In 2D simulations, the transducer offset was varied between 0 and 200 μm in 5 μm steps, where 0 μm corresponds to the symmetrical placement of the transducer. The acoustic pressure was solved at a range of frequencies between 0.7 and 1.7 MHz. The admittance of the transducer and the channel’s maximum acoustic pressure were evaluated. The maxima of both admittance and acoustic pressure were observed for all the offset values at 1.136  MHz. The admittance plot obtained from the 2D simulations is illustrated in Fig. 7. The plot showing the maximum pressure in the chip as a function of different offset values is presented in Fig. S8 in the supplementary material.

**FIG. 8.**
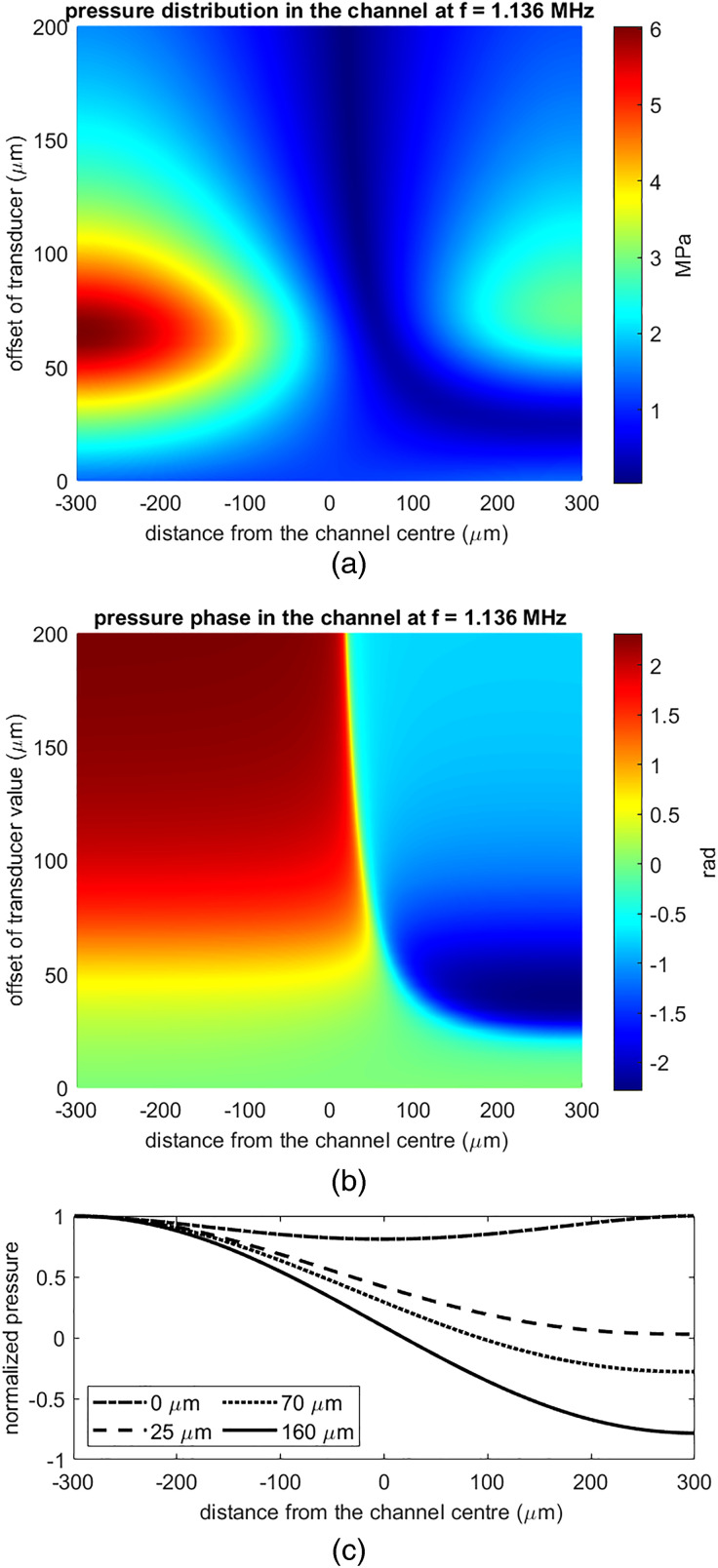
The variation of (a) magnitude and (b) phase of the acoustic pressure in the channel, evaluated at 1.136  MHz for different offset values. The normalized pressure distribution in the channel at 1.136 MHz is given in (c).

The effect of symmetry breaking is clear in Fig. 7. The offset placement of the transducer creates additional peaks on both sides of the main peak and increases the peak values. Figure 8 presents the variation in the magnitude and phase of the acoustic pressure in the channel as a function of the transducer offset evaluated at 1.136 MHz. In Fig. 8(c), the normalized pressure distribution in the channel is given for offset values of 0, 25, 70, and 160 μm, again demonstrating the onset of the pressure node and the effect of increasing offset. In Fig. 8(c), the acoustic pressure is evaluated at the central line of the channel.

Figure 8 demonstrates that breaking the symmetry via including an offset is necessary to obtain a pressure node in the channel. A pressure node starts to form in Figs. 8(b) and 8(c) as the offset gets larger than 25 μm. Around the offset value of 70 μm, the maximum peak pressure is observed in the channel, as well as the phase variation gets its strongest. While these values are derived from a simplified study, Fig. 8 demonstrates that a symmetrically placed transducer cannot generate the pressure node perpendicular to the transducer, while there is also an optimal value for the offset for the most efficient operation. For the same offset values, 2D pressure and phase plot in the cross section of the channel are given in Fig. S10 and section “Pressure field across the channel’s cross section” in the supplementary material.

#### 2. 3D simulations

Although 2D simulations were useful for demonstrating that symmetry breaking is necessary to generate a standing wave with a pressure node in the channel, they cannot explain how the oil droplets are trapped in certain spots in Fig. 5(b). With the purpose of understanding this trapping behavior, 3D simulations of the whole microfluidic chip were carried out. First, the location of the transducer on the chip was identified as [x,y]=[0.1079,0.1593] mm, where the origin is taken as the geometrical center of the chip surface (see Fig. S7 and section “Determining exact location of transducer” for further details in the supplementary material). The trapping location of the oil droplets depends largely on the location of the transducer. To study the potential effects of the small mistakes in determining the location of the transducer on the trapping locations, a sensitivity study was performed. The details can be found in the section “Sensitivity analysis for computer simulations” in the supplementary material.

As the transducer is offset from the center, the acoustic field inside the channel is calculated for a frequency window to compare the experimental droplet locations with simulations. For such a comparison, the first option is to add particle tracing physics to the model and solve for droplet trajectories in a separate study. The alternative and quicker method is to evaluate the Gor’kov potential for each acoustic field, which is already solved. Given that the droplets will be trapped at the potential minima, the Gor’kov potential is a quicker and equally reliable way of determining the trapping locations of the droplets.^19,20^

**FIG. 9.**
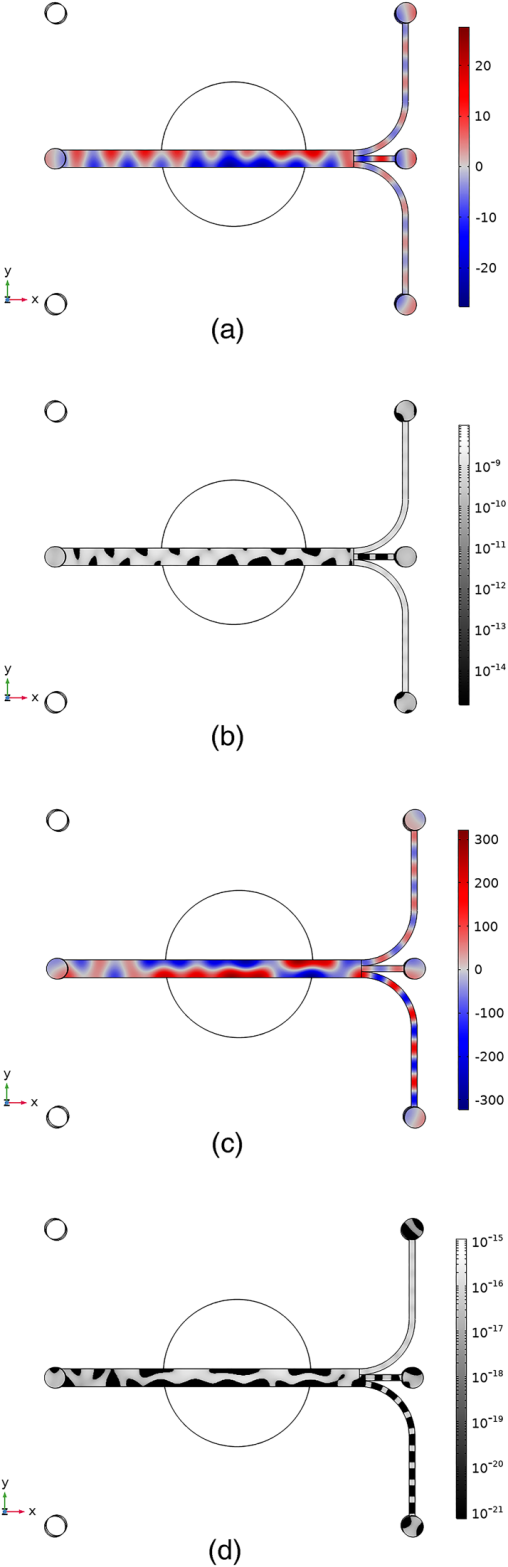
Calculated acoustic pressure (kPa) (a) and (c) and Gor’kov potential (nJ) (b) and (d) at 1.1313 MHz (a) and (b) and 1.21  MHz (c) and (d) for hexadecane oil droplets. Note the potential is given in the log scale to highlight the trapping locations. In both cases, the transducer is placed at the offset location at [x,y]=[0.1079,0.1593]  mm.

While the transducer is placed at the offset location at [x,y]=[0.1079,0.1593] mm, the acoustic field inside the chip was first solved for the frequency window of 1.1–1.3 MHz, with a step of 5 kHz. The corresponding calculated Gor’kov potentials in the simulations were used to identify the matching pattern with the experimental particle trajectories. The admittance maximum was found at 1.21 MHz, making it the so-called most suitable frequency of operation. The distribution of the Gor’kov potential further led to narrowing down the search to a smaller frequency window. As the second step, a more in-depth frequency stepping was carried out between the frequency range of 1.125–1.135 MHz, with steps of 1 kHz. The final step of frequency stepping was carried out between 1.13 and 1.132 MHz, with steps of 50 Hz, leading to the identification of the acoustic field at 1.1313 MHz, where we found a match between the Gor’kov potential and the experimental trapping pattern. The acoustic field and corresponding Gor’kov potential for hexadecane at 1.1313 and 1.21 MHz are given in Fig. 9. The Gor’kov potential is useful for obtaining the trapping positions, since the oil droplets are trapped at the positions of the minimum Gor’kov potential. The Gor’kov potential is calculated according to Eq. (2). The standing waves are also formed diagonally across the channel’s cross section in different offset location of the transducer. The corresponding pressure and phase distribution plots from 2D and 3D simulations are shown in section “Pressure field across the channel’s cross section” in the supplementary material. The results clearly indicate that when the transducer is symmetrically placed with the channel, the pressure distribution in the channel is the so-called trivial solution, where there is no phase difference in the acoustic pressure in the channel.

Comparison of Figs. 9(a) and 9(c) highlights the critical differences between operating at resonance [Fig. 9(c)] and non-resonance [Fig. 9(a)]. In Fig. 9(c), there is a single, albeit wavy, pressure node in the middle of the channel. Under this condition and a continuous flow, solid particles would be concentrated in the midchannel, while the liquid droplets would move toward the edges. Such a case enables the separation of particles from droplets. A more critical difference is that the non-resonant condition in Fig. 9(a) already has an order of magnitude lower pressure amplitude than the resonance conditions in Fig. 9(c).

A careful look at the Figs. 9(a) and 9(b) reveals that the chip is not excited in a half-wave resonance mode for the channel. The pressure node’s serpentine-like pattern is neither parallel nor perpendicular to the flow direction inside the main channel. However, the pressure nodes and anti-nodes are distributed among the channel length in the outlet channels, resulting in pressure nodes/anti-nodes perpendicular to the flow. The patterns of the experimental trapping locations in Fig. 5 overlap with the corresponding Gor’kov potential for hexadecane droplets of 6 μm diameter in Fig. 9(b). This overlap demonstrates the possibility of trapping droplets when the transducer is displaced from its center and at a non-resonance frequency, which is also confirmed by computer simulations.

It is worth noting that even though the trapping patterns overlap, the frequencies at which similar behavior is observed differ between the simulations and experiments. The maximum admittance in the experiments was at 1.18 MHz, while in the computer simulations, it was observed at 1.21  MHz. Similarly, the experiments were carried out at 1.25  MHz, and similar trapping locations were observed at 1.13  MHz in the computer simulations. This difference can be caused by deviations in the material parameters, which usually result in shifts in the frequency response of systems. In addition, the computer simulations assume a perfect interface between the transducer and the glass chip. Nevertheless, the trapping patterns in simulations and experiments match even though there are uncertainties in the material parameters and not all the possible physical interactions were included in the computer simulations.

## V. CONCLUSIONS

Almost every study in the literature investigates the systems in resonance conditions and aims to improve the performance of acoustofluidic particle separators. Contrary to this approach, this study shows that operating at a non-resonant frequency with a minimal off-center location of the transducer result in a predictable behavior of oil droplets in acoustofluidics. The 2D simulations showed that symmetry breaking is necessary to obtain a pressure node in the channel, and it can be observed only after a certain offset placement of the transducer. The experimental trapping patterns of hexadecane droplets were replicable in computer simulations. Furthermore, not only is free-flow separation still possible, but the experimental and simulation results also show that the trapping patterns can act against the flow direction. This further suggests that such conditions can lead to applications for filtering and concentration with single-input-single-output chip geometries. This is relevant in applications concerning *in-vivo* manipulation of biological matter within living tissues.^59^ In such applications, theoretical resonant frequencies may not be achieved due to limitations such as the fixed size of the chamber and the inability to place the piezoelectric transducer in the desired location.

## SUPPLEMENTARY MATERIAL

Further details of the following experimental and simulation procedures along with the corresponding results can be found in the supplementary material.
∙Monodisperse emulsion fabrication, acoustic experimental results, particle tracking velocimetry, acoustic velocity of oil droplets, particle image velocimetry, determining exact location of transducer, sensitivity analysis of computer simulations, maximum pressure based on 2D simulations, pressure field across the channel’s cross section, mesh convergence analysis, fabrication of the microfluidic chip, effects of secondary acoustic radiation force, and effects of streaming force.

## Data Availability

The data that support the findings of this study are openly available in 4TU repository at https://doi.org/10.4121/c7e58257-87d9-4ea9-b6e8-5cf9570e9cd3, Ref. 60.
